# Advances in Anti-Cancer Drug Development: Metformin as Anti-Angiogenic Supplemental Treatment for Glioblastoma

**DOI:** 10.3390/ijms25115694

**Published:** 2024-05-23

**Authors:** Siddharth Shah, Hadeel M. Mansour, Tania M. Aguilar, Brandon Lucke-Wold

**Affiliations:** 1 Department of Neurosurgery, University of Florida, Gainesville, FL 32608, USA; siddharth.dr99@gmail.com (S.S.);; 2 College of Medicine at Chicago, University of Illinois, Chicago, IL 60612, USA

**Keywords:** glioblastoma, metformin, antineoplastic, novel therapies for glioblastoma, neurosurgery, anti-cancer drugs

## Abstract

According to the WHO 2016 classification, glioblastoma is the most prevalent primary tumor in the adult central nervous system (CNS) and is categorized as grade IV. With an average lifespan of about 15 months from diagnosis, glioblastoma has a poor prognosis and presents a significant treatment challenge. Aberrant angiogenesis, which promotes tumor neovascularization and is a prospective target for molecular target treatment, is one of its unique and aggressive characteristics. Recently, the existence of glioma stem cells (GSCs) within the tumor, which are tolerant to chemotherapy and radiation, has been linked to the highly aggressive form of glioblastoma. Anti-angiogenic medications have not significantly improved overall survival (OS), despite various preclinical investigations and clinical trials demonstrating encouraging results. This suggests the need to discover new treatment options. Glioblastoma is one of the numerous cancers for which metformin, an anti-hyperglycemic medication belonging to the Biguanides family, is used as first-line therapy for type 2 diabetes mellitus (T2DM), and it has shown both in vitro and in vivo anti-tumoral activity. Based on these findings, the medication has been repurposed, which has shown the inhibition of many oncopromoter mechanisms and, as a result, identified the molecular pathways involved. Metformin inhibits cancer cell growth by blocking the LKB1/AMPK/mTOR/S6K1 pathway, leading to selective cell death in GSCs and inhibiting the proliferation of CD133+ cells. It has minimal impact on differentiated glioblastoma cells and normal human stem cells. The systematic retrieval of information was performed on PubMed. A total of 106 articles were found in a search on metformin for glioblastoma. Out of these six articles were Meta-analyses, Randomized Controlled Trials, clinical trials, and Systematic Reviews. The rest were Literature review articles. These articles were from the years 2011 to 2024. Appropriate studies were isolated, and important information from each of them was understood and entered into a database from which the information was used in this article. The clinical trials on metformin use in the treatment of glioblastoma were searched on clinicaltrials.gov. In this article, we examine and evaluate metformin’s possible anti-tumoral effects on glioblastoma, determining whether or not it may appropriately function as an anti-angiogenic substance and be safely added to the treatment and management of glioblastoma patients.

## 1. Introduction

Glioblastomas (GBMs) are the most prevalent primary central nervous system (CNS) neoplasms, accounting for around 30–40% of these malignancies [1,2]. They are derived from neural stem cells, earlier believed to originate from glial cells [2,3]. The incidence is about 6 per 100,000 people reported annually [2]. GBM has a poor prognosis with a mean survival of 15–18 months post-diagnosis [2,4]. The World Health Organization (WHO) developed the Classification of Tumors of the Central Nervous System to describe these tumors accurately. They categorized them into four groups based on their level of aggressiveness [2,4]. The great majority of all primary CNS tumors are high-grade gliomas (WHO grade III and IV). The most frequent and malignant form of glioma is glioblastoma multiforme (GBM, WHO grade IV glioma). It is estimated that it makes up about 15% of diagnoses [2,4,5]. Surgery and radiotherapy are all common treatments for GBM which have not been able to improve outcomes and survival significantly [6]. 

In an attempt to improve survival in GBM and discover novel therapies for GBM, many researchers have looked into common therapies used in practice. One of them is metformin, which is 1,1-dimethyl biguanide hydrochloride [7]. Metformin has been the most frequently prescribed glucose-lowering drug for the past 60 years. It is now the first-line medicine for people newly diagnosed with type 2 diabetes mellitus (T2DM) in several clinical recommendations [8,9]. Metformin usage may also reduce cancer risk and improve cancer prognosis [9]. Metformin may impact tumorigenesis indirectly, through the systemic lowering of insulin levels, and directly, by inducing energy stress; however, these effects require additional exploration [10]. Its possible role in tumor pathogenesis has recently been identified [11,12,13]. The use of metformin has been linked to improved overall and progression-free survival in individuals with high-grade gliomas [14]. Metformin repurposing as a cancer therapy is already being tried in several clinical studies for glioblastoma which are mentioned in Table 1. 

## 2. Mechanism of Action of Metformin on Cancer Cells, Antineoplastic Action, and Edema Reduction

Metformin is widely recognized as the frontline treatment for type 2 diabetes mellitus [15]. Its mechanisms of action extend beyond glycemic control, encompassing the inhibition of gluconeogenesis, enhancement of insulin sensitivity, and modulation of cellular metabolism [16,17,18,19]. Over the years, metformin has demonstrated inhibitory effects on the growth, survival, and metastasis of various cancers, including lung, breast, colon, prostate, endometrial, melanoma, leukemia, and pancreatic cancer [20]. Investigators have suggested a reduced cancer incidence and mortality in individuals treated with metformin, warranting its potential as an adjunctive therapy in cancer management [21]. Metformin has garnered significant attention for its potential as an anti-cancer agent in the treatment of glioblastoma (GBM), a highly aggressive brain neoplasm known for largely being resistant to treatment, prone to recurrence, and associated with a devastating prognosis [22]. Interestingly, studies have shown that metformin enhances the anti-tumor efficiency of chemotherapeutic agents in vitro and in vivo [23,24], and recently, metformin has been shown to selectively kill cancer stem cells with minor adverse effects [22,25,26,27]. 

One of metformin’s key mechanisms of action, enabling its anti-hyperglycemic effect, is through the activation of adenosine monophosphate-activated protein kinase (AMPK). AMPK, a regulator of multiple cellular processes, deploys a wide range of significant effects on cancer, including the inhibition of cellular proliferation by halting mitotic cell division [20,28]. This loss of cellular proliferation in cancer cells is by way of the inhibition of cyclin D1 proto-oncogene. It is a critical regulator of the G1 to S-phase transition of the cell cycle and is frequently found overexpressed in many cancers [28,29,30]. Similarly, tumor suppressor genes play a major role in the abnormal proliferation of tumor cells. The loss or inactivation of the P53 gene is the most frequently altered, especially in highly aggressive cancers [31,32]. Through the activation of AMPK, metformin can induce P53 phosphorylation and activation, promoting cell cycle arrest to allow for DNA repair and apoptosis [33]. This process inhibits potential cancer cell invasion and metastasis.

The overexpression of the mammalian target of rapamycin (mTOR), a kinase that regulates cell growth and metabolism, in cancer, is associated with tumor progression, cancer drug resistance, and a worse prognosis [30]. Metformin inhibits mTOR through AMPK-dependent and -independent mechanisms, contributing to its anti-cancer effects [34]. The AMPK-dependent inhibition of mTOR is regulated by the ratio of adenosine monophosphate (AMP) to adenosine triphosphate (ATP). As metformin enters cancer cells, it makes its way to the mitochondrial membrane, where it inhibits complex I of the electron transport chain, resulting in a decreased ATP-to-AMP ratio [35,36]. This decrease in cellular energy is sensed by AMPK, resulting in its activation, followed by a cascade of downstream effects including P53 and cyclin D1 regulation as aforementioned [35]. The AMPK-independent inhibition of mTOR is achieved by way of DNA damage-inducible transcript 4 (REDD1) activation and by metformin’s direct inhibition of mTOR. REDD1 hypoxia-induced signaling inhibits mTOR expression. Metformin treatment has been shown to significantly increase the expression of REDD1, which in turn inhibits mTOR, thereby affecting the further advancement of cancer, such as that seen in glioma [37,38,39]. Metformin inhibits mTORC1 indirectly by activating AMPK, which in turn inhibits the mTORC1 pathway [9]. This inhibition leads to reduced protein synthesis and cell proliferation. mTORC2 is primarily involved in regulating cell survival, metabolism, and cytoskeletal organization. One of its key roles is activating Akt (also known as protein kinase B) through phosphorylation at serine 473 [9]. There is some evidence suggesting that metformin might indirectly affect mTORC2 activity, but this mechanism is less understood. Metformin’s influence on mTORC2 could be secondary to its effects on cellular energy status and AMPK activation. Akt activation is a critical step for cell survival and growth. Metformin has been shown to decrease Akt activity, although the exact mechanism is not fully elucidated [40]. It may involve AMPK-dependent and AMPK-independent pathways. In vitro studies often utilize high dosages of metformin (millimolar range) to effectively suppress Akt activation [9]. For example, doses of 1–5 mM are frequently utilized in cell culture models to produce significant impacts on Akt phosphorylation. In Vivo Research and Clinical Achievability: Animal Models: In vivo investigations in animal models, such as mice, have employed doses of 500 mg/kg/day to achieve effective plasma concentrations capable of inhibiting Akt [39]. These levels result in substantial systemic exposure, with corresponding human doses of about 2000 mg/day. Human Studies: The maximal tolerable dosage of metformin in humans is around 2000–2500 mg/day, which is routinely used to treat type 2 diabetes [39,40]. At these levels, achieving intra-tumoral concentrations high enough to directly inhibit Akt may be difficult due to pharmacokinetic restrictions such as drug distribution and blood–brain barrier permeability. While routine dosage often results in systemic plasma concentrations in the micromolar range, reaching millimolar concentrations within tumors, particularly brain tumors, is more difficult. Dosing Strategies: Higher dosages of metformin or combination methods with other medications to improve its delivery to the tumor location may be required. However, the safety and acceptability of such techniques must be carefully assessed [39,40].

In general, SGK (serum and glucocorticoid-induced protein kinase)-, AKT (serine/threonine-protein kinase)-, and PKC (protein kinase C)-related pathways are suppressed upon mTOR inhibition. This results in reduced ion transport, increased apoptosis, and an increase in cytoskeletal reorganization, respectively [41].

Metformin demonstrates anti-cancer effects, including decreased proliferation, cell cycle arrest, apoptosis induction, and the promotion of autophagy and pyroptosis. Autophagy induced by metformin occurs through an AMPK-dependent mechanism that leads to ULK1 phosphorylation and indirect mTOR inhibition [42,43,44]. Pyroptosis, known for its role in pathogen clearance, is distinct from other forms of cell death due to its inflammatory activation of inflammasomes and caspases, resulting in cell swelling, membrane pore formation, and the release of pro-inflammatory cytokines and cellular contents into the extracellular space, triggering an inflammatory response [35,45,46]. Metformin’s capacity to induce cellular pyroptosis is being increasingly investigated as a novel therapeutic strategy for various cancers. It operates by activating sirtuin 1, an NAD+-dependent deacetylase, through AMPK, leading to downstream NF-kB expression [35,46].

Metformin has the potential to impact not only the progression of cancer but also the severity of its symptoms, including brain edema. Brain tumors disrupt the blood–brain barrier (BBB) due to the hypoxic environment created by rapidly growing cancer cells. This disruption leads to increased levels of vascular endothelial growth factor (VEGF) and subsequent vascular changes that can disrupt the endothelial tight junctions that make up the BBB [42,47,48]. Studies have shown that metformin, when administered orally, can cross the blood–brain barrier and be widely distributed throughout the central nervous system. This distribution allows metformin to reduce vasogenic edema by activating the AMPK pathway in BBB endothelial cells, leading to enhanced barrier tightness [42,49]. Since temozolomide (TMZ) already has a restricted ability to pass through the blood–brain barrier, any additional reduction may actually make it less effective. To optimize TMZ penetration, it may be necessary to carefully monitor the time and dosage of metformin and TMZ. One method to improve clinical tactics may be to time TMZ delivery such that it reaches optimal brain concentrations before starting metformin or to use metformin in a way that minimizes its influence on TMZ penetration.

## 3. Glioblastoma Stem Cell Target for Metformin

Metformin’s anti-cancer activity includes mechanisms that have been found to target stem cells, such as glioma stem cells (GSCs), which have the capacity for proliferation, undergo intrinsic regeneration, and differentiation [42,48,50]. The presence of GSCs in aggressive glioma forms, such as glioblastoma, may be the underlying cause of treatment resistance and recurrence following therapy [42,51]. Metformin offers a potential way to combat this issue by way of the intracellular targeting of pathways such as AMPK. Studies indicate that upon AMPK activation, the Forkhead Box O3 (FOXO3) transcription factor is inhibited, resulting in a loss of GSC differentiation and likely preventing its transformation to fully malignant cancer cells [25,52].

**Figure 1:**
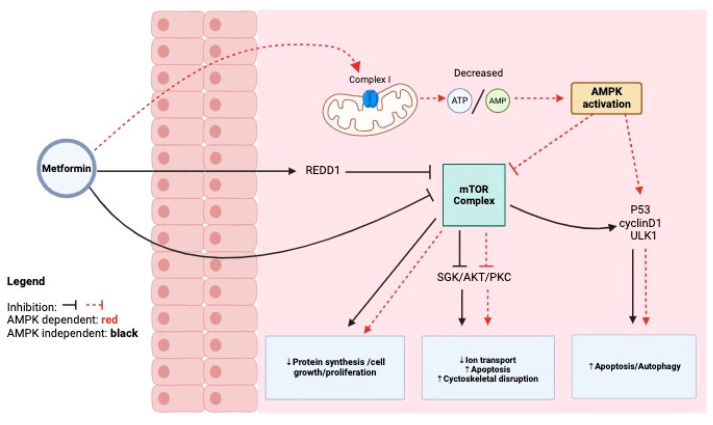
Glioblastoma stem cell target and mechanism of action.

Novel biguanide derivatives are being developed, using metformin as a blueprint, for pharmacologically targeting GSCs. Metformin exerts anti-tumor effects in GSCs by inhibiting chloride intracellular channel-1 (CLIC1), a protein known for promoting GSC survival, proliferation, and migration [22,53]. While there are still contradictory results from translational studies regarding metformin’s anti-cancer efficacy in GSC subsets, Barbieri et al. provide insight into the importance of CLIC1 as a major determinant of cancer stem cell activity in glioblastoma. To address this determinant, powerful and selective treatments for GBM and other CLIC1-dependent tumors require biguanide-based compounds [54]. This process is well depicted in Figure 1.

## 4. Metformin for High-Grade Glioblastoma+Trials

Metformin, a frontline treatment for type 2 diabetes, has attracted significant attention as a potential therapeutic agent for glioblastoma (GBM), a highly aggressive brain tumor. Epidemiological studies have hinted at a reduced cancer incidence and mortality rate in individuals treated with metformin, suggesting its promise as an adjunctive therapy in glioma management [55,56,57,58,59,60].

Adeberg et al. observed longer progression-free survival among diabetic patients with primary GBM who received metformin treatment [59,61]. Similarly, Seliger et al. demonstrated improved overall and progression-free survival in patients with high-grade gliomas, particularly WHO grade III tumors, under metformin therapy [62]. Notably, this association was more pronounced in grade III gliomas compared to grade IV tumors.

A study on the use of metformin plus temozolomide-based chemotherapy as adjuvant treatment for malignant gliomas found that there was a significant important difference between TMZ alone and TMZ plus metformin arms in six of the cases [61].

In a 2019 retrospective report, Seliger et al. suggested a potential correlation between metformin treatment and better survival outcomes in patients with grade III gliomas, possibly linked to a higher percentage of IDH mutations in WHO grade III gliomas [62]. Further analysis by Seliger et al. involving a larger cohort of GBM patients failed to establish a significant relationship between metformin monotherapy and overall survival or progression-free survival, underscoring the complexity of the relationship between metformin use and glioma prognosis [62,63].

Studies show that when metformin is used with temozolomide for individuals with newly diagnosed GBM, it not only has a good safety profile but may also have therapeutic advantages [64,65,66]. Several clinical trials are currently underway to explore the efficacy of the combination of metformin and temozolomide in treating certain cancer types. Additionally, Welch et al. reported that metformin monotherapy was among the most important predictors of survival in their retrospective analysis of GBM patients [67].

Amro H. Mohammad et al.’s recent study presented the advantage of metformin treatment for survival in patients with GBM that have the methylation of the MGMT promoter [68]. These findings suggest a potential role for metformin in personalized treatment strategies for GBM patients with specific molecular characteristics.

The use of metformin was linked to prolonged survival in patients with tumors featuring a methylated O6-methylguanine DNA methyltransferase gene (MGMT) promoter. Cox regression analysis revealed that metformin was associated with a reduced risk of death at 2 years among patients with glioblastoma harboring a methylated MGMT promoter [67]. Table 2 summarizes the trials on high-grade glioma treated with metformin and other common therapies.

## 5. Adjuvant Therapy Metformin and Temozolomide for Newly Diagnosed Glioblastoma

Research indicates that metformin not only demonstrates a favorable safety profile in non-diabetic individuals but also exhibits potential therapeutic benefits when used in conjunction with temozolomide for newly diagnosed glioblastoma patients [64,65,66]. Preclinical studies have shown promising results regarding metformin’s efficacy in GBM, both as a monotherapy and in combination with standard chemotherapeutic agents like temozolomide (TMZ). Recent research has elucidated the synergistic interaction between metformin and TMZ, resulting in enhanced cytotoxicity and improved treatment outcomes in GBM models [68]. Based on these preclinical studies and trials, it has been proposed that the combination of MET and TMZ may be an effective treatment for some cancer types.

In addition, MET treatment in combination with TMZ induces a synergistic anti-tumoral response in glioma cell lines. A combined treatment approach utilizing metformin and temozolomide presents promising potential benefits, particularly in addressing the challenge posed by MGMT. MGMT, a DNA repair protein, counteracts the alkylating effect of temozolomide by removing alkyl groups from the O6 position of guanine in DNA. The methylation status of the MGMT promoter serves as a critical predictive biomarker for glioblastoma responsiveness to temozolomide. Active MGMT expression enables tumor cells to mitigate the effects of temozolomide by facilitating DNA repair and promoting tumor growth. Conversely, metformin demonstrates anti-cancer properties by suppressing cancer cell cycling and proliferation, enhancing cancer cell apoptosis, and reducing cell viability. Importantly, metformin has shown its ability to effectively inhibit MGMT expression and its downstream signaling, potentially reversing temozolomide resistance associated with MGMT [69,70].

Previous studies across various cancer cell lines have established that metformin, either alone or in combination with other DNA-damaging agents, can downregulate MGMT expression and enhance treatment efficacy. Therefore, combining temozolomide with metformin holds promise for enhancing temozolomide’s effectiveness and increasing the susceptibility of previously chemoresistant cells to temozolomide [70].

Furthermore, the combination of metformin and temozolomide may confer protection against reactive oxygen species (ROS) injury in tumor cells. Studies have demonstrated an additive increase in cancer cell death following radiotherapy when temozolomide is combined with metformin, mediated by an elevation in ROS levels. Additionally, research exploring the synergistic effect of metformin with ionizing radiation in cancer treatment has revealed metformin’s capacity to induce apoptosis post-radiation, thereby augmenting cell death in cancer cells [69,70].

Previous studies have shown that the combination treatment of TMZ and MET synergistically inhibits growth and induces cell apoptosis in both U87 GSCs and U251GSCs, accompanied by a reduction in mTOR, S6K, and 4EBP1 signaling. MET effectively inhibits TMZ-induced AKT activation. Although combining the two drugs leads to powerful AMPK activation, this synergy is not definitive. This synergy was confirmed in vivo by a significant reduction in tumor burden and significantly prolonged median survival of tumor-bearing mice after combined drug treatment compared to treatment with either drug alone [70,71].

As we navigate the uncharted waters of glioblastoma therapy, the convergence of TMZ and MET beckons a new era of precision oncology. As clinicians and researchers stand on the cusp of groundbreaking discoveries, the imperative to translate benchside triumphs into bedside victories has never been more urgent. Together, let us forge ahead, armed with the knowledge that the synergy between TMZ and MET holds the key to unlocking the elusive cure for glioblastoma.

## 6. Results

### 6.1. Metformin’s Mechanism of Action in Cancer Cells

Metformin inhibits cancer progression primarily through the AMPK pathway, impacting cellular metabolism and promoting anti-proliferative effects across various cancer types. Studies have shown metformin’s efficacy in reducing cellular proliferation, inducing cell cycle arrest, and enhancing apoptosis, especially in glioblastoma through mechanisms that include the modulation of the mTOR/S6K1 pathway and direct action on glioblastoma stem cells (GSCs). While the general pharmacokinetics of metformin are well characterized, specific data for glioma patients are limited. Research in this area is crucial to optimize the use of metformin for potential anti-tumor effects in brain malignancies, ensuring efficacy and safety.

### 6.2. Impact on Edema and Brain Tumor Environment

Metformin’s potential in managing brain edema and altering the microenvironment of brain tumors has been substantiated through its regulatory effects on the blood–brain barrier and inflammatory responses, significantly reducing vascular permeability and mitigating edema-associated symptoms.

### 6.3. Adjuvant Therapy with Temozolomide

Clinical trials have explored the combination of metformin and temozolomide, indicating enhanced therapeutic outcomes in newly diagnosed glioblastoma patients. This combination has been effective in overcoming temozolomide resistance, potentially through metformin’s inhibition of the MGMT gene, which is a crucial player in chemotherapy resistance. The AMPK activation process has the potential to block the mTOR pathway, which plays a role in cell growth and proliferation. This inhibition encourages cell cycle arrest and apoptosis, which might intensify TMZ’s cytotoxic effects on glioma cells. Glioma cells can become more vulnerable to TMZ-induced cell death by decreasing pro-survival pathways, which is achieved by lowering insulin/IGF-1 signaling. By targeting glioma stem cells, metformin may reduce tumor recurrence and enhance the overall effectiveness of TMZ and radiation therapy. Metformin can increase oxidative stress within tumor cells. This increased oxidative stress can make glioma cells more vulnerable to the DNA-damaging effects of TMZ, thereby enhancing its cytotoxic impact. Improved immune responses can work alongside TMZ to more effectively target and kill glioma cells.

### 6.4. Effectiveness in High-Grade Glioblastoma

The adjunctive use of metformin in high-grade glioblastoma has been associated with improved survival rates, particularly in patients with MGMT promoter methylation. The synergistic effects of metformin with standard therapies could pave the way for its integration into current glioblastoma treatment protocols.

## 7. Discussion

Our study has reinforced the role of metformin not just as an anti-diabetic drug but as a potent anti-cancer agent, offering a dual advantage in glioblastoma treatment—targeting both cancer cells and the tumor environment. Metformin’s mechanism of action through the AMPK pathway and its effects on the mTOR/S6K1 axis suggest comprehensive anti-tumoral activity, from reducing cell viability to modifying the tumor’s edema and microenvironment. Clinical trials focusing on the combined use of metformin and temozolomide have highlighted significant improvements in treatment outcomes, which are promising for the integration of metformin into glioblastoma management strategies. However, the variability in responses based on genetic differences such as MGMT promoter methylation status calls for a personalized approach to therapy.

Further research should aim at understanding the differential responses based on genetic markers and expanding the scope of metformin’s efficacy in other aggressive forms of cancer. The exploration of metformin’s full potential in oncology could significantly alter the landscape of cancer treatment, especially for conditions as challenging as glioblastoma.

## 8. Conclusions

In conclusion, metformin shows antineoplastic mechanisms against glioblastoma cells by increasing apoptosis and autophagy and inhibits cancer cell growth by blocking the LKB1/AMPK/mTOR/S6K1 pathway. Therefore, it is proposed to hold substantial promise as an adjunctive therapy in glioblastoma treatment due to its extensive anti-cancer properties and favorable safety profile. Its ability to enhance the efficacy of temozolomide and potentially reverse drug resistance offers a novel avenue for improving outcomes in glioblastoma patients. Future clinical trials should continue to focus on optimizing dosing regimens and identifying patient subgroups who are most likely to benefit from this combined treatment approach, thereby harnessing metformin’s full therapeutic potential. Even while many trials have produced some extremely intriguing results, they do not provide a complete picture of the intricacy of the tumor process and should be supplemented with relevant research conducted on animal models or patient observations. It indicates that more research will be required to determine the precise features of metformin’s impact on the metabolism of high-grade gliomas. A detailed comprehension of this drug’s mechanism of action may lead to the discovery of novel medications for neoplasm therapy. Overall, we believe that our study highlights metformin as a possible therapeutic option in GBM patients.

## 9. The Limitations of This Study

This study faces several limitations, including a homogeneous patient sample that might not represent the diverse demographic characteristics of the broader glioblastoma population, potentially limiting the generalizability of the findings. The optimal dosing regimen for metformin as an adjunctive therapy to glioblastoma remains undefined, which could affect the consistency and efficacy of treatment outcomes. Additionally, the study’s short follow-up period restricts the evaluation of long-term effects and survival benefits, while the clinical settings of the trials may not accurately reflect real-world environments. The influence of MGMT promoter methylation on treatment response introduces further variability, as metformin’s effectiveness could differ significantly between methylated and unmethylated tumor subtypes. Moreover, the study design did not fully account for potential confounders such as concurrent medications and comorbidities, which could skew the results. Statistical challenges due to small sample sizes in certain subgroup analyses might lead to underpowered findings, and the lack of a detailed exploration into the mechanistic pathways of metformin’s action leaves some theoretical aspects of its anti-cancer properties unverified. Finally, the absence of randomization or blinding could introduce bias, affecting the reliability of the conclusions drawn.

## Figures and Tables

**Table 1:** Currently registered active and completed clinical trials for using metformin in glioblastoma or malignant brain tumors.

Title	Country	Population (n)	Outcomes/Objectives	Trial No.
Temozolomide, Memantine Hydrochloride, Mefloquine, and Metformin Hydrochloride in Treating Patients With Glioblastoma Multiforme After Radiation Therapy	USA	144	The purpose of this study was to determine the safety and tolerability of temozolomide (TMZ) in combination with metformin (metformin hydrochloride) (MFRMN), mefloquine (MFLOQ), and/or memantine (memantine hydrochloride) (MEMTN) in patients receiving adjuvant therapy after completing external beam radiotherapy (XRT) in combination with chemotherapy for newly diagnosed glioblastoma multiforme.	NCI-2011-03038
Metformin, Neo-adjuvant Temozolomide, and Hypo- hypo-accelerated radiotherapy Followed by Adjuvant TMZ in Patients With GBM	Canada	50	It is expected that the proposed study treatment will improve the median survival from current values of 20 months (current MUHC neoadjuvant Phase 2 data) to 25 months. This means an improved outcome of 25%. Using one-tailed statistics, with a power of 0.8 and an alpha of 0.05, the sample size for this Phase II trial will be 50 patients.	NCT02780024
Bioavailability of Disulfiram and Metformin in Glioblastomas	Sweden	3	Does the drug get there? Does the drug perform what it is intended to perform? To improve the chances of clinical success, there is a need for the rational and intelligent selection of potential drugs in future trials. This is an initiative for analyzing the tumor concentration of preoperatively administered repurposed drugs.	NCT03151772
A Pilot Study of Ketogenic Diet and Metformin in Glioblastoma: Feasibility and Metabolic Imaging	USA	36	Not published.	NCT04691960
Study on Low Dose Temozolomide Plus Metformin or Placebo in Patient With Recurrent or Refractory Glioblastoma	South Korea	81	A comparison of progression-free survival obtained from the progression-free survival curve.	NCT03243851
Phase 2, Open-label, Single-arm Study on the Use of Metformin as Adjunctive Therapy in High-grade Glioma	Italy	25	The clinical trial will be single-arm to evaluate the biological activity and effects of metformin in combination with TMZ in patients with GBM. The efficacy at the recommended dose (RD) of metformin in patients with GBM.	NCT05929495
Oxidative Phosphorylation Targeting In Malignant Glioma Using Metformin Plus Radiotherapy Temozolomide	France	640	Progression-free survival (PFS) estimated by the RANO (Response Assessment in Neuro-Oncology) criteria.	NCT04945148
Treatment of Recurrent Brain Tumors: Metabolic Manipulation Combined With Radiotherapy	Israel	18	The short-term implementation of the metabolic intervention (i.e., combined diet and metformin therapy) before, during, and after hypofractionated (2 weeks) radiation therapy is expected to increase tolerability, increase compliance, and avoid the chronic metabolic complications associated with extreme carbohydrate-restriction diets.	NCT02149459

**Table 2:** Clinical trials on high-grade glioma treated with metformin being studied.

Country	Study ID	Population (n)	Objectives/Outcomes	Treatment
France and Italy	NCT04945148	640	The investigators have observed in vivo (a reduction of >50% of tumor growth) and hypothesize that metformin could be specifically efficient to treat up-front patients affected by OXPHOS+ GBM, in association with the standard first-line treatment with radiotherapy and temozolomide (RT-TMZ).	Metformin (1500–3000 mg daily) plus radiation and temozolomide
Israel	NCT02149459	18	To enhance the radiosensitivity of recurrent brain tumors through metabolic reprogramming induced by a low-carbohydrate diet and metformin therapy.	Metformin, radiation, and low carbohydrate diet
Canada	NCT02780024	50	This trial seeks to validate the adjunctive use of metformin with established GBM therapies, hypothesizing that its metabolic effects will synergistically enhance the efficacy of standard treatment protocols, thereby significantly improving patient survival outcomes.	Metformin and neoadjuvant temozolomide followed by combined radiation and temozolomide
USA	NCT04691960	36	The purpose of the study is to evaluate the tolerability of a ketogenic diet in conjunction with metformin and whether maintaining and the diet with metformin will have any effect on participants. Participants will prepare their own meals with the help of a nutritionist. Participants will continue on treatment as long as they are responding to therapy and not experiencing unacceptable side effects.	Metformin (ramp up to 850 mg three times daily) and ketogenic diet
USA	NCT05183204	33	The purpose of this study is to assess the safety of Paxalisib while maintaining a ketogenic diet (a high-fat, low-carbohydrate diet) and metformin (a drug approved by the Food and Drug Administration to treat type 2 diabetes) and to see what effects it has on glioblastoma.	Metformin (ramp up to 850 mg three times daily as tolerated), and ketogenic diet
USA	NCT01430351	144	From the study entry, the median survival was 21 months, and the 2-year survival rate was 43%. Memantine, mefloquine, and metformin can be combined safely with TMZ in patients with newly diagnosed glioblastoma.	Metformin (dose not specified), mefloquine, memantine, hydrochloride, hydrochloride, and temozolomide
South Korea	NCT03243851	81	Although the metformin plus temozolomide regimen was well tolerated, it did not confer a clinical benefit in patients with recurrent or refractory GBM.	Metformin (ramp up to 2000 mg daily) and low-dose temozolomide
